# Progress of Epidemics in Thirty-Eight Towns and Districts

**Published:** 1857-01

**Authors:** 


### PROGRESS OF EPIDEMICS.

### LOCAL REPORTS OF EPIDEMIC AND ENDEMIC DISEASES

*During the Months of September, October, and November,* 1856.

| Place. | County. | Lat. | Long. | Observer. |
|---|---|---|---|---|
| St. Mary, Scilly | Cornwall - | 49.50 N. | 6.18 W. | J. G. Moyle, Esq. |
| Teignmouth - | Devonshire - | 50.32 N. | 3.29 W. | W. C. Lake, Esq. |
| Canterbury - | Kent - - | 51.17 N. | 1. 4 E. | G. Rigden, Esq. |
| Chatham - | Kent - - | 51.21 N. | 0.14 E. | F. J. Brown, M.D. |
| Wandsworth - | Surrey - - | 51.28 N. | 0. 7 W. | G. E. Nicholas, Esq. |
| Putney - - | Surrey - - | 51.28 N. | 0. 8 W. | R. H. Whiteman, Esq. |
| Up. Holloway | Middlesex - | 51.32 N. | 0.03 E. | W. B. Kesteven, Esq. |
| Wanstead - | Essex - - | 51.32 N. | 0. 2 W. | F. Collins, M.D. |
| Swansea - | Glamorgansh. | 51.38 N. | 3.50 W. | W. H. Michael, Esq. |
| Saffron Walden | Essex - - | 52. 3 N. | 0.12 E. | T. Spurgin, Esq.<br>H. Stear, Esq. |
| Bedford - - | Bedfordshire - | 52. 8 N. | 0.51 W. | T. H. Barker, M.D. |
| Sharnbrook - | Bedfordshire - | 52.12 N. | 0.40 W. | R. S. Stedman, Esq. |
| Newpt. Pagnell | Buckinghams. | 52.10 N. | 0.42 W. | G. O. Rogers, Esq. |
| Wellingbro' - | Northamptons. | 52.20 N. | 0.40 W. | B. Dulley, Esq. |
| Beccles - - | Suffolk - - | 52.25 N. | 1.48 E. | W. E. Crowfoot, Esq. |
| Thetford - | Norfolk - - | 52.26 N. | 0.45 E. | H. W. Bailey, Esq. |
| Dudley - - | Staffordshire - | 52.30 N. | 2.10 W. | J. H. Houghton, Esq. |
| Barrowden - | Rutland - | 52.34 N. | 0.38 W. | H. J. Swann, Esq. |
| Wisbeach - | Cambridgesh. | 52.39 N. | 0. 5 E. | W. H. Hole, Esq. |
| East Dereham | Norfolk - - | 52.40 N. | 0.57 E. | J. Vincent, M.D. |
| Pontesbury - | Shropshire - | 52.43 N. | 2.50 W. | Wm. Eddowes, Esq. |
| Nottingham - | Nottinghamsh. | 52.50 N. | 1.10 W. | T. Robertson, M.D. |
| Burton-on-Trt. | Staffordshire - | 52.53 N. | 1.53 W. | S. Thomson, M.D. |
| Oswestry - | Shropshire - | 52.53 N. | 2.59 W. | P. Cartwright, Esq. |
| Wrexham - | Denbighshire - | 53. 2 N. | 3. 1 W. | E. Williams, M.D. |
| Hawarden - | Flintshire - | 53.11 N. | 3. 2 W. | T. Moffat, M.D. |
| Lincoln - | Lincolnshire - | 53.12 N. | 0. 5 W. | S. Lowe, Esq. |
| Alford - - | Lincolnshire - | 53.15 N. | 0. 6 E. | R. U. West, M.D. |
| Staveley - | Derbyshire - | 53.15 N. | 1.20 W. | G. B. Thorpe, Esq. |
| Gainsborough | Lincolnshire - | 53.23 N. | 0.47 W. | D. Mackinder, M.D. |
| Liverpool - | Lancashire - | 53.24 N. | 2.59 W. | Thos. Bickerton, Esq. |
| Warrington - | Lancashire - | 53.25 N. | 2.32 W. | C. N. Spinks, Esq. |
| Wigan - - | Lancashire - | 53.32 N. | 2.33 W. | W. I. Cox, Esq. |
| Bolton - - | Lancashire - | 53.35 N. | 2.19 W. | W. H. Pendlebury, Esq. |
| York - - | Yorkshire - | 53.58 N. | 1. 3 W. | W. Procter, Esq. |
| Wst. Auckland | Durham - | 54.45 N. | 1.40 W. | G. Todd, Esq. |
| Rothbury - | Northumberld. | 55.25 N. | 1.50 W. | E. C. Summers, Esq. |
| Lerwick - | Shetland Isles | 60. 9 N. | 1. 8 W. | G. W. Spence, M.D. |

### QUARTERLY STATEMENT—No. VIII.

[*The dates denote that the disease appeared in the weeks then ending.*]

SCARLET FEVER.

Teignmouth..Oct. 17, Nov. 21
Canterbury..Oct. 24-31, all November
Chatham..Sept. 5-19, Oct. 10, 24 31
Wandsworth..Sept. 26, all Oct., Nov. 7, 28
Putney..November 28
Upper Holloway..September 5
Swansea..All Sept., Oct. 10-17, 31, November 14, 28
Saffron Walden..Sept. 19, Oct. 3-10, 24, Nov. 7, 21
Bedford..Sept. 12
Sharnbrook..Sept. 12-26, all October, Nov. 7-14
Dudley..Sept. 12-19, Oct. 10, 24-31, Nov. 7, 21

Wisbeach..Sept.5-12,Oct.10-31,Nov.7
East Dereham..Every week
Nottingham..Sept. 12,26, Oct. 17-24
Burton-on-Trent..October 24
Oswestry..Oct. 31, Nov. 7-21
Wrexham..Oct. 10-31, Nov. 7-21
Liverpool..Every week
Warrington..Every week
Bolton-le-Moors..Sept. 19-26, Oct. 17-[31, Nov. 21-28
York..Nov. 21-28

MEASLES.

Teignmouth..Sept. 5, 19, all Oct., [Nov. 7-14
Canterbury..Oct. 10
Chatham..Sept. 12, Oct. 10, 24, Nov. [7, 21
Wandsworth..Sept. 5
Putney..October 31, November 7
Upper Holloway..November 14-28
Swansea..September 5, 19
Bedford..Every week
Wellingborough..Sept. 26, Oct. 3-17
Beccles..October 3, 10
Barrowden district..September 5-19
Wisbeach..Oct. 17-31, all Nov.
East Dereham..November 28
Pontesbury..Sept. 5, 12, Nov. 21-28
Nottingham..Oct. 3, Nov. 14-21
Oswestry..October 10-24
Hawarden..November 21
Gainsborough..October 24, 31
Liverpool..Every week
Warrington..November 14-28
Bolton-le-Moors..Oct. 3-17, Nov. 7-21
York..September 26, October 17
West Auckland..September 5-19

SMALL-POX.

Swansea..November 28 (doubtful)
Wellingborough..Oct. 3-17,Nov.21-28
Dudley..Sept. 5, 12-26, all October, November 7, 28
Wisbeach..Oct. 17-31, Nov. 7-14
Pontesbury..Sept. 19, Oct. 17-31
Nottingham..October 3
Liverpool..Every week
Bolton-le-Moors. October 24, 31
West Auckland..September 12-26
Lerwick..Every week

HOOPING COUGH.

Teignmouth..Oct. 3, Nov. 7
Canterbury..Every week except last
Wandsworth..Sept. 26, all Oct. & Nov.
Putney..Sept. 26, all Oct., Nov. 7-14
Upper Holloway..September 5
Swansea..Every week except last
Bedford..Oct. 10-31, all Nov.
Wellingborough..November 21-28
Thetford..September 19
Dudley..October 31
Barrowden district..Sept. 26, Oct. 3, [24, Nov. 14
Wisbeach..Sept. 5, 12
Pontesbury..Nov. 14-28
Nottingham..All Sept., Oct. 3, 10, 24, 31; November 7, 21, 28
Oswestry..Oct. 10-31, Nov. 7-21
Staveley..Sept. 19, 26, all Oct. & Nov.
Liverpool..Every week
Warrington..Every week
Bolton-le-Moors..Sept. 26, Oct. 10, 24, 31, all November.
York..Sept. 19, Oct. 3, 17, 24, Nov. 7
West Auckland..All Oct., Nov. 7-21

CROUP.

Canterbury..October 17
Chatham..October 10
Swansea..October 17
Newport Pagnell..November 21
Beccles..November 7-14
Thetford..October 17
Nottingham..Sept. 12, 19, Oct. 31, November 7-14
Wrexham..Oct. 10, 17, Nov. 14-28
Lincoln..November 7
Liverpool..Sept. 12, 26, Oct. 3, 17, 31, November 14-28
Warrington..November 21
Bolton-le Moors..Sept. 19, Oct. 17, 24
York..Sept. 12, Oct. 10-24, Nov. 14

CATARRH.

Scilly Islands..September 5
Teignmouth..Every week expt.Oct.17
Chatham..Sept. 19, 26, all Oct. & Nov.
Wandsworth..All Sept., Oct. 10, 24, November 7, 21, 28
Putney..Oct. 17, 24, all Nov.
Upper Holloway..Sept. 12, Nov. 14-28
Wanstead..Sept. 12, Nov. 14
Swansea..September 19, October 10
Saffron Walden..Sept. 12, all Nov.
Bedford..September 12, 19
Sharnbrook..Sept. 5-19, Oct. 10-31, all November
Newport Pagnell..Oct. 3, 10, 24, 31, all November
Wellingborough..Sept. 26, all Oct. & [Nov.
Beccles..Sept. 26, Oct. 3, 10, 24, 31, Nov. 21-28
Thetford..Sept. 5, 12, 26, Oct. 10, 17, [31, all Nov.
Wisbeach..Sept.26, Oct. 3, 31, all Nov.
East Dereham..All November
Nottingham..Oct. 10, 31, Nov. 7-14
Burton-on-Trent..Oct. 10, 17, 31
Oswestry..September 5, 19
Wrexham..November 14-28
Staveley..All November
Gainsborough..Every week
Liverpool..Every week
Warrington..Sept.19,26,Oct.3,allNov.
Wigan..Oct. 24, 31, Nov. 7-14
Bolton-le-Moors..Sept.26, Oct. 10-31, [Nov. 14-28
Rothbury..All Nov.
Lerwick..Oct. 31, all November

INFLUENZA.

Teignmouth..Oct. 3, 31, Nov. 7
Chatham..Sept. 26, all Oct. and Nov.
Wandsworth..Sept.12, Oct.31, Nov,21
Wanstead..Sept.12-26, all Oct.& Nov.
Saffron Walden .Oct. 10, 17, all Nov.
Bedford..November 14-28
Sharnbrook..Oct. 17-31, all Nov.
Newport Pagnell..Sept. 19, 26, Oct. 17-31, all November
Wellingborough..All November
Beccles..Sept. 5, 19, Oct. 3, 17, 24
Thetford..All Sept., Oct. 3, 10, 24, 31
Dudley..Oct. 10, Nov. 14 [all Nov.
Nottingham..Sept. 5, 12, 26, Oct. 24, November 7
Oswestry..Sept. 26, Oct. 3-24
Wrexham..Oct. 24, 31, all Nov.
Hawarden..October 3
Lincoln..October 10-24
Alford..Septembe 19
Staveley..October 31, November 7, 14
Liverpool..Sept. 12, 26, Oct. 10, 31, November 14-28.
Warrington..November 14-28
Bolton-le Moors..Oct. 31, Nov. 7-21
York..Sept.12,19, Oct.10,24, Nov.7-21
West Auckland..Oct. 10-31, all Nov.
Lerwick..Oct. 31, all Nov.

ERYSIPELAS.

Scilly Islands..September 12
Canterbury..Oct. 10, Nov. 7-21
Chatham..Sept. 12, 19, Oct. 3, 10, 24, November 14, 21
Wandsworth..Sept. 19, Nov. 7
Putney..Sept. 19, 26, Oct. 3, 10
Wanstead..October 10-24
Swansea..Sept. 26, Oct. 10
Saffron Walden..September 26
Bedford..November 7-14
Sharnbrook..November 7
Newport Pagnell..Sept. 12, Oct. 10, November 14, 28
Wellingborough..Oct. 24, 31, Nov. 28
Thetford..Sept. 12, 19, Oct. 3, 31,
Dudley..Oct. 17 [Nov. 7, 14, 28
Pontesbury..All November
Nottingham..All Sept., Oct. 3, 17, November 7, 14
Oswestry..Sept. 5, 12, Oct. 10 24
Hawarden..October 31
Alford..November 14
Staveley..All Sept., Oct. 3, 10
Gainsborough..November 28
Liverpool..Sept. 12-19, Oct. 3, 17-31, November 7, 21-28
Warrington..Sept. 5, 19, 26, Oct. 17, November 14-28
Bolton-le-Moors..Sept. 26, Oct. 3, 17
York..November 21

CHOLERA.

Chatham..September 5, 19
Newport Pagnell..Sept. 19, Oct. 10
Dudley..September 5
Barrowden..Sept. 5 (English)
Liverpool..Oct. 3, 10 (English)

AGUE.

Canterbury..October 3-17
Chatham..Sept. 5-19, Oct. 3-24, Nov.
Wanstead..Sept. 5, Oct. 17 [14, 28
Bedford..October 3, November 14-28
Sharnbrook..October 31
Thetford..November 21
Barrowden..September 26
Wisbeach..Sept. 5-19, Oct. 24, 31, all
Alford..Oct. 10-31, Nov. 7-21 [Nov.
Warrington..November 14-28

REMITTENT FEVER.

Teignmouth..Sept. 5, 12, Oct. 3, 24, 31
Wandsworth..November 7
Putney..October 17-31, November 7
Upper Holloway..September 12
Swansea..Sept. 5, 12, Oct. 10, 24, 31,
Saffron Walden..Sept. 5-19 [Nov. 7
Sharnbrook..September 5-19
Newport Pagnell..Sept. 26, Oct. 10, 17, November 28
Beccles..Sept. 5, 12, Nov. 7, 21
Thetford..September 19
Dudley..September 19, October 31
Barrowden district..Sept. 12-26, Oct. 3
Wisbeach..Sept. 26, all Oct.
Nottingham..Sept. 5-19, Oct. 3, 24, 31, Nov. 7, 21, 28
Oswestry..Sept. 19, all Oct., Nov. 7-14
Alford..Sept. 12, 26, Oct. 3
Staveley..Sept. 12-26, Oct 3, 10
Liverpool..Sept. 12-19, Oct. 3, 17, 24,
Wigan..October 10-24 [Nov. 14-28
Bolton-le-Moors..Sept. 5, 19, 26, Oct. 24, 31, Nov. 14
York..October 24, 31, November 7
West Auckland..September 5, 12

DIARRHŒA.

Scilly Islands..September 12
Teignmouth..All Sept. and October, November 14, 28
Canterbury..Every week except last
Chatham..All Sept., Oct. 3-24, Nov.
Wandsworth..Every week [14 28
Putney..All Sept., Oct. 3-17, Nov. 21
Upper Holloway..All Sept., Oct. 3
Wanstead..All Sept., Oct. 3-24
Swansea..All Sept., Oct. 17, 31
Saffron Walden..Sept., 12 26, all Oct.,
Bedford..Every week [Nov. 21, 28
Sharnbrook..Every week expt. Oct. 31
Newport Pagnell..Every week except November 21
Wellingborough..Oct. 10-31, Nov. 28

Beccles..All Sept., Oct. 17-31, Nov. [21, 28
Thetford..Every week
Dudley..Every week except Sept. 12
Barrowden..Sept. 5-19, Oct. 10
Wisbeach..All Sept., Nov. 21, 28
East Dereham..All Sept., Oct. 17, all [Nov.
Pontesbury..All September
Nottingham..All Sept. and October, November 21, 28
Burton-on-Trent..Every week except [Nov. 7
Oswestry..All Sept., Oct. 3-24
Wrexham..Oct. 31, Nov., 7,14
Hawarden..Sept. 12-26, all Oct.&Nov.
Alford..Oct. 10-31, Nov. 7
Staveley..October 3, 10
Gainsborough..Sept. 5, 12, Oct. 17-31, all November
Liverpool..Every week
Warrington..Sept. 12-26, all Oct.
Wigan..October 31, all November
Bolton-le-Moors..Sept. 12-26, Oct. 10
Rothbury..Sept. 19, Nov. 14
Lerwick..All November

DYSENTERY.

Canterbury..November 28
Chatham..Sept. 5-19, Oct 3, 10
Putney..Sept. 12, Nov. 28
Upper Holloway..Oct. 31, Nov. 7-14
Wanstead..Sept. 5, 12, Nov. 21
Swansea..September 19
Bedford..October 3, 10
Newport Pagnell..Sept. 19, Oct. 10,17
Beccles..Oct. 24, 31, Nov. 7, 14
Thetford..November 14
Dudley..September 26
Wisbeach..Sept. 26, Oct. 3, 10
Nottingham..All Sept. & Oct.
Oswestry..September 12, 19
Gainsborough..September 5, 12
Liverpool..Sept. 12-19, Oct. 24-31, November 21-28
Warrington..All Sept., Oct. 3, 17, 24
Bolton-le-Moors..All Sept., Oct. 3, 10

TYPHUS.

Scilly Islands..November 7-21
Teignmouth..October 3, 17
Canterbury..Sept. 19, 26, all Oct., Nov. 7-21
Chatham..Sept. 5, 19, 26, Oct. 3, 10, [24, 31, Nov. 14, 21
Wandsworth..Sept. 12-26, Oct. 24, Nov. [21-28
Putney..Sept. 12, all Nov.
Bedford..All Sept., Oct. 3,31, Nov. 7,14
Sharnbrook..October 17
Beccles..October 31, November 7
Thetford..Sept. 12, all Oct. & Nov.
Dudley..Every week except Oct. 10
Wisbeach..All October, November 7
Pontesbury..Oct. 31, all Nov.
Nottingham..Sept. 5, 12, Nov. 14
Oswestry..Oct. 10-31, Nov. 7-21
Wrexham..November 28
Lincoln..All September, October 3
Staveley..Oct. 10, 31, all Nov.
Gainsborough..Every week
Liverpool..Every week
Warrington..September 5
Bolton-le-Moors..Sept. 5, 19, all Oct., November 14 [7, 14
West Auckland..All Sept. & Oct., Nov.
Rothbury..October 24, 31
Lerwick..October 10

PUERPERAL FEVER.

Chatham..November 21, 28
Bedford..November 21
Newport Pagnell..October 3
Dudley..September 12, 19
Nottingham..November 21
Staveley..All November
Warrington..October 17

CARBUNCLE.

Scilly Islands..October 31
Canterbury..October 31
Chatham..November 7
Putney..September 19
Wanstead..November 7
Swansea..Oct. 3, 10, Nov. 7
Saffron Walden..October 3, 31
Newport Pagnell..September 5, 12
Wellingborough..November 28
Thetford..Sept. 12, Oct. 3, 10, 24, [Nov. 14, 21
Dudley..Sept. 12
Barrowden..September 26
Burton-on-Trent..Sept. 5, Nov. 14
Oswestry..Sept. 12-26, Oct. 3-24
Wrexham..October 24, 31
Alford..November 7
Staveley..Every week
Liverpool..Oct. 17-31, all Nov.
Wigan..Sept. 19, 26, Oct. 3
Bolton-le-Moors..October 3, 10, 31

CONTINUED FEVER.

Swansea..All September

MUMPS.

Liverpool..Oct. 4 (1 case fatal)

---

*St. Mary's, Scilly.*—Mr. Moyle says: "We have had five deaths this quarter in a population of 3,600. There were no deaths in September; in which month there were only a few cases of catarrh, and one (mild) of erysipelas of the head and face. The mean temperature for the month was 66°, and the weather very fine. The rain gauged 2·61 inches. Two deaths occurred in October; one from paralysis in an old man aged seventy-six; the other in a person aged seventy-five, from diseased heart. The case of carbuncle recovered. The weather was fine in October; the mean temperature was 60°. The rain gauged 3·29 inches. In November there were three deaths: one in an old woman aged seventy-two, from carcinoma of the breast and axilla; one in a man aged thirty-two, from phthisis; and one from typhus in a young man aged nineteen, one of a large family, living in rooms but seldom ventilated; the mother is now ill with the same disease. The weather was fine: the rain gauged 1·3 inch."

*Teignmouth.*—Mr. Lake writes: "From September 1st to the 8th the weather was fine, but colder than it had been at the close of August; from the 9th to the 17th it was mostly fine, warm, and pleasant; from the 17th to the end of the month it was mild, rainy, and cold; the barometer between the 22nd and 29th being continuously depressed. The first week in October was also rainy, but close and warm; from September 17th to October 6th 3·486 inches of rain fell; from October 8th to November 10th there alternated short periods of cold dry and moist warm weather; from November 11th to the 15th the weather was very cold for the time of year, but fine; from the 15th to the 28th it was fine, mild, and pleasant. Between October 9th and November 9th the barometer, on three days only, fell below 30 inches. It rained, on one day only between October 16th and November 8th. From October 16th to November 24th, the total rain fall only measured 0.446 inch. November was, throughout, a fine and bright month; on the 28th frost set in. The principal disorder of the quarter was measles. Of forty-two cases that came under my notice, nineteen were uncomplicated; in one there were convulsions; two were accompanied with pneumonia; eight with bronchitis; four with diarrhœa; three with bronchitis and diarrhœa; two with ulcerative stomatitis; one was followed by impetigo; one by lichen; and one by hooping-cough. Two deaths were registered from measles in the town during the quarter."

*Canterbury.*—Mr. Rigden says: "Scarlet-fever is becoming epidemic in this city and neighbourhood. Hooping-cough continues. Several cases of erysipelas, particularly affecting the head and face, have been brought under treatment. A few cases of ague have shown themselves. Diarrhœa, although neither malignant nor very general, has continued to prevail. Fever has also continued; and a few cases of carbuncle have occurred. In the month of September deaths were registered as resulting from diarrhœa, catarrh, and mumps; in October from diarrhœa, scarlet-fever, and erysipelas; in November from fever, scarlet-fever, diarrhœa, and dysentery."

Mr. Haffenden furnishes the subjoined returns, drawn up by eight observers for the Epidemiological Committee of the East Kent and Canterbury Medical Society.

"*Abstract of Meteorological Observations for the Autumn Quarter*, 1856.

| | *Sept.* Deg. | *Oct.* Deg. |
|---|---|---|
| Highest temperature in the day time............ | 63 | 61 |
| Lowest.................................... | 47 | 35 |
| Mean ..................................... | 54·13 | 51·41 |
| Highest reading of barometer.................. | 30·16 | 30·39 |
| Lowest.................................... | 28·80 | 29·55 |
| Mean ..................................... | 29·683 | 30·02 |
| Number of days on which rain fell.............. | 16 | 12 |
| Amount of rain in inches ...................... | 3·84 | 2·21 |

Direction of the wind (the number indicates the number of days the wind prevailed)..*September*—N. 3, NE. 1, E. 3, SE. 3, S. 1, SW. 10, W. 3, NW. 6; *October*—N. 2, NE. 6, E. 7, SE. 3, S. 5, SW. 4, NW. 3.

(No return furnished for November.)

*Abstract of the returns of Zymotic diseases during the Autumn Quarter*, 1856.

"The number of cases which have been returned by the eight observers during the autumn quarter has been 162; and rather more than a third of these were diarrhœa.

"Scarlatina. An increase of two cases in each month has been noticed. One case proved fatal in September, it was of the malignant form. Two deaths occurred in October; one a few days after the appearance of anasarca; the other in a child three years old, who had convulsions thirteen hours after the appearance of the eruption.

"Measles, which have for some months been on the decline, seem to have disappeared altogether this quarter.

"Small-pox. Not a single case has been returned during the quarter.

"Hooping-cough furnished sixteen cases, the greater number occurring in October; only one in November; not one of them proved fatal.

"Diarrhœa. Fifty-eight cases have been reported; more than half of these were in the month of September; five only in the last month. They were chiefly ordinary cases; one was fatal; in this patient the disease continued for three weeks, never very severe, but suddenly causing great prostration, syncope, and death

"Fever shows a slight decrease this quarter. Fifteen cases have been returned, one of them ending fatally. Pneumonia being the cause.

"Ague also shows a slight decrease, five cases only being returned.

"Mumps. In September five cases were reported, and one in October. Death was the result of one, from great prostration, seven days after the commencement of the disease.

"Erysipelas and erythema. Eleven cases have been returned, all of which recovered."

*Chatham.*—Dr. Brown says: "Stomatitis prevailed in September, but no deaths occurred from it. Two cases of metria came under my notice in the end of November. I attribute them to paludal causes, as marsh affections have been rife throughout the quarter, and I ascertained that a connection existed between the metria that prevailed in the first quarter of 1853 and the ague class of diseases. The two cases occurred near one another, on the river side of the High-street of Rochester. It is remarkable that aguish attacks after delivery are not uncommon on this side of the street, whilst they are unknown on the opposite side. The same is the case with ague in the non-puerperal state; it affects only one side of the street. One of the cases of metria proved fatal, and was thus registered:—'child-birth,' seven and a half days; 'metria,' five and a half days. The symptoms were—rapidity of pulse, sweating, vomiting, and exhaustion. There was no abdominal complication until the last day, when there was dysuria and some pain. The second case is recovering. It consisted in mild uterine inflammation (uterine phlebitis, I believe), succeeded by pyæmia; diarrhœa was a troublesome symptom in this case. The treatment of the first case was stimulant; that of the second consisted in leeching, and the use of mercury with opium, whilst the strength was supported by wine and brandy. Both cases were attended by the same accoucheur; other women residing on the opposite side of the street, attended at the same time, even on the same day, escaped. During the past three months several cases of typhoid fever occurred, in which large quantities of blood passed from the bowels; they all recovered favourably. No case of the species typhus occurred in these towns that I myself witnessed. One case is stated to have come from London; death occurred about the eleventh day."

*Wandsworth.*—Mr. Nicholas says, "Scarlet-fever and hooping-cough, which commenced simultaneously towards the end of September, have been the prevailing epidemics of the last quarter. Although diarrhœa is shown in the table to have been of weekly occurrence, the cases were isolated, and readily amenable to treatment. Many of the cases of scarlet-fever, however, commenced with diarrhœa. Scarlet-fever has now nearly subsided; but hooping-cough still continues, attacking principally infants at the breast, and the very young. From this circumstance, and its occurrence at this season of the year, the disease has had a very early tendency to the supervention of capillary bronchitis, and pneumonia. Some cases, in infants, have occurred as pneumonia primarily, its pathognomonic signs becoming developed only on the subsidence of inflammatory action. Cattle have been entirely free from epidemic disease."

*Putney.*—Mr. Whiteman says: "At the early part of last quarter diarrhœa of a mild form prevailed extensively in the undrained portions of this district. Hooping-cough was somewhat prevalent, but of a mild type, during the whole of October and the first two weeks of the following month. For a length of time this district has been unusually free from diseases of the zymotic class, and the rate of mortality has for months been exceedingly low. The following is an extract from one of my weekly reports to the District Board of Works, furnished in my capacity of Medical Officer of Health for this locality.

" 'The last half yearly summary of sickness and mortality as given in the medical relief book of my own sub-district (Putney and Roehampton), is of a most satisfactory character: and I consider that it well illustrates the benefits resulting from improved sanitary regulations. The poor attended by me in sickness and accident, during the half year just expired (29th September), have been 363 in number. The deaths from all causes during the same period have been—from diarrhœa (ages three and eight months), 2; from infirmity of age (ages ninety-seven and eighty years), 2; from paralysis (age seventy), 1; total deaths, 5.'

" Deducting the two deaths from natural decay, the low rate of mortality here exhibited, extending over a period of six months, is somewhat remarkable, and is certainly without a parallel in the returns I have furnished during the nine years of my official connexion with the Wandsworth and Clapham Union."

*Wanstead.*—Dr. Collins states that the cases of influenza affected chiefly very young children.

*Swansea.*—Mr. Michael says: "The weather of this quarter has been markedly fine, beyond the average of the season, and the public health has been also good, in an equal ratio. Although scarlet-fever has now been for many months epidemic, there have been very few severe cases; and almost every case has proceeded to a favourable termination. Hooping-cough prevails extensively, but not in a severe form."

*Saffron Walden.*—Mr. Spurgin says: "The quarter has been remarkably healthy, and the season generally dry. Rheumatism and neuralgic affections of the face have, however, been prevalent, particularly in October, when the weather for the season was unusually foggy. Vegetation has been protracted till a later period than usual from the absence of severe frosts."

*Bedford.*—Dr. Barker writes: "Measles and diarrhœa have prevailed throughout the quarter, and hooping-cough during the latter half. In many cases measles and hooping-cough have existed concurrently in the same subject, and, in some localities, measles and diarrhœa. These concurrent diseases, especially in early infancy, have proved fatal in several instances. Diarrhœa, although generally controllable, has in several cases assumed a dysenteric character. The dense fogs in this neighbourhood in the early part of November, and the frequent and sudden changes of temperature, have been followed by a large accession of disease, particularly influenza, ague, bronchitis, and neuralgic affections. Several cases of paralysis have also come under my notice. The potato is considerably diseased in many places in the neighbourhood."

*Sharnbrook.*—Mr. Stedman says that scarlet-fever was prevalent all the quarter, but in a very mild form. It appeared principally among the poorer classes; no fatal cases occurred.

*Beccles.*—Mr. Crowfoot says that mumps and neuralgia were prevalent during this quarter; cerebral congestions and apoplectic attacks were frequent in October.

*Thetford.*—Mr. Bailey writes: "The specific epidemics of last quarter have entirely left us, excepting one case of hooping-cough which occurred in the third week of September. Throughout the month the temperature was high, and the atmospheric pressure deviating but little, until the last ten days, when its fluctuation was great: the lowest point on the 28th being 28·80, at which time the greatest amount of rain fell. This state of temperature, together with the little wind, produced fewer diseases than usually occur at this period.

Catarrh, diarrhœa, and influenza were the most prevalent complaints. Two cases of erysipelas came under notice; their progress was arrested by the application of nitrate of silver. A fatal case of typhus gravior occurred in a lad of bad habits and delicate constitution, in a poor family; but no other case took place in the village. Remittent fever occurred in a poor man sent to the Union House from the Fens; he quickly recovered by the change of residence. During the last week of September several cases of pleurisy occurred in labouring men, arising from exposure to the wet and winds; these required very active treatment; bronchitis in children came under notice from the same cause. Rheumatism, both acute and chronic, have been generally under medical treatment, almost throughout the year; but more so within the last few days of this month. One case of carbuncle upon the loins occurred in a female; it was of large size, producing much constitutional irritation and suffering, but ultimately did well. The casual complaints during the month were—tic douloureux, menorrhagia, dyspepsia, hepatitis, dropsy, infantile convulsions, and some cutaneous diseases.

"In the early part of October, which was wet with a high temperature, overcast days and a dew point averaging the temperature, more disease occurred. Fever assuming a low type seemed more epidemic than usual, attacking families isolated from each other in different villages; several in one family were attacked at the same time. The disease commenced with some diarrhœa, which was neglected at first, producing extreme exhaustion, and followed by febrile symptoms running a long course, with various congestions: those families who were the subjects of the fever were in low circumstances, being deprived of many necessaries of life. I consider it epidemic and not infectious. In many instances only one in a large family, under the same circumstances, was afflicted; and other families at a distance, where no communication whatever took place, were attacked at the same time. In this sanitary age, cesspools, privies, bad water, etc., etc., have been supposed to be the cause of fever, but these causes have existed in the same localities for very many years, without such fever being produced. Diarrhœa was still very prevalent, and might be said to be epidemic; scarcely a day passed without a new case. One case of croup ended suddenly fatal in a child after a few hours attack. Pleurodynia was more prevalent this month, affecting suddenly those who are not exposed to the weather. Some cases of consumption, ending fatally, occurred. The casual diseases were syphilis, abortion, anasarca, cancer uteri, atrophy, fistula in ano, and some cutaneous diseases.

"The great vicissitudes both in atmospheric pressure and temperature during November, especially in the last week from the excessive cold, produced many cases of pneumonia, influenza, and catarrhal affections. Diarrhœa still continued very prevalent, attacking all grades of society, and, unless early attended to, led on to consecutive fever. Erysipelas was certainly epidemic in this locality ; every week produced fresh cases, attacking the face and head, and avoiding the body. In one instance a child at the breast became affected, the mother having the disease in the face. Two cases of carbuncle occurred, one in a clergyman, and the other in a tradesman, both situated at the nape of the neck ; under the usual treatment both recovered. Low adynamic or typhoid fever still continued in the surrounding parishes, assuming the same type and commencing with neglected diarrhœa; this disease was confined to the very poor, several in a family being seized at the same time, and their means of support being very scanty. It was not confined to low and damp localities, but attacked those living in dry and well ventilated houses, and not holding any intercommunication. The cinchonine plan of treatment has no advantage over the ordinary mode of treatment—the disease runs its course and leaves the patient emaciated and powerless, and frequently with boils in various parts. Free ventilation has been insisted upon, with proper support by mild nutriments. Of many cases under my care, two have terminated fatally. The casual diseases of the month under notice—hæmorrhoids, syphilitic eruptions, sciatica, hydrocele, inguinal hernia (reducible), and some cutaneous diseases."

*Record of Deaths in Twenty-one Parishes (population 9,574) from the Registrar's Book.*

| *September.* | | *October.* | | *November.* | |
|---|---|---|---|---|---|
| Diarrhœa | 2 | Croup | 1 | Consumption | 5 |
| Debility | 3 | Bronchitis | 2 | Atrophy | 3 |
| Inflammation of chest | 1 | Dropsy | 1 | Diseased bladder | 1 |
| Consumption | 2 | Paralysis | 1 | Congestion of brain | 1 |
| Atrophy | 2 | Diseased heart | 1 | Scarlatina | 1 |
| Premature birth | 1 | Decay of nature | 3 | Bronchitis | 1 |
| Congestion of brain | 1 | Consumption | 4 | Diseased heart | 1 |
| Bronchitis | 1 | Cancer | 1 | Diarrhœa | 1 |
| Decay of nature | 1 | | | Fever | 2 |
| Diseased bladder | 1 | | | Premature birth | 1 |
| Dropsy | 1 | | | Decay of nature | 2 |
| Fever | 1 | | | | |
| Diseased liver | 1 | | | | |
| Apoplexy | 1 | | | | |
| | 19 | | 14 | | 19 |

*Dudley.*—Mr. Houghton says: "Scarlet-fever has prevailed to a small extent during the last quarter. Small-pox has also prevailed throughout the quarter, and some fatal cases have occurred. Diarrhœa and fever are probably never altogether absent from the town, and will no doubt continue till the Board of Health shows more rigour than is at present manifested; on the whole, however, the quarter has been a healthy one, and the deaths have been below the average. Jaundice has occurred to a considerable extent amongst children, *not infants.* There has been but very little disease amongst cattle."

*Barrowden.*—Mr. Swann writes: "Measles abated at the beginning of the quarter; I have had no cases since. The fever cases were chiefly confined to Morcott; out of six cases, one died. Generally speaking, with the exception of those few cases, the district has been remarkably healthy. Within this last fortnight there has been a good deal of sore throats, coughs, and inflammations of the respiratory organs, arising from keen strong north-east and north-west winds. In the district there has also been, and still is, much disease (inflammation) of lungs among cattle. Potatoes, after the heavy rains, became much diseased."

*East Dereham.*—Dr. Vincent writes: "Many of the cases of scarlet-fever were rapidly fatal from coma: in comparatively few cases were the tonsils greatly affected; still fewer had abscess afterwards, but almost all suffered from dropsy; and a few died from pneumonia supervening upon the dropsy. Many of the cases (even of the most rapidly fatal) occurred in cottages of the better kind. In almost all cases the disease could be traced to communication with other families already infected."

*Nottingham.*—Dr. Robertson says: "The quarter has been marked by very peculiar changes in the atmosphere, which have unquestionably exerted considerable influence upon the state of the public health. September, usually one of our finest months, has this year been excessively wet; and it was most fortunate that in the south and midland counties the harvest was secured before the principal fall of rain occurred. The greatest fall occurred on the 28th (two and a half inches). It continued wet until October 16th; after which date but little rain fell to the end of the month. In November rain fell on six days, and a heavy fall of snow took place on the 26th; followed by very severe frost to the end of the month. The mean temperature of September was 2°, that of October 3°, and that of November 3° below the average. The potato crop this season is extensively diseased; and root crops (especially turnips and mangel-wurzel) are expected by the farmers to suffer severely from the intense frost. Horses and cattle have suffered chiefly from diseases of the respiratory organs. Pleuro-pneumonia, of a low type, has been especially prevalent towards the conclusion of the quarter.

"The accompanying plan shows the number of deaths which occurred from epidemic and endemic diseases during the quarter; and I have also appended, by way of comparison, a similar return for the corresponding period in 1855.

| | QUARTERS ENDING | |
|---|---|---|
| | *Nov.* 30, 1855. | *Nov.* 28, 1856. |
| Scarlet fever | 2 | 0 |
| Measles | 0 | 1 |
| Small-pox | 0 | 0 |
| Hooping cough | 1 | 7 |
| Croup | 2 | 4 |
| Catarrh | 0 | 0 |
| Influenza | 0 | 0 |
| Erysipelas | 1 | 0 |
| Cholera | 0 | 0 |
| Ague | 0 | 0 |
| Remittent fever | 0 | 2 |
| Diarrhœa | 37 | 20 |
| Dysentery | 1 | 2 |
| Typhus | 1 | 5 |
| Puerperal fever | 0 | 1 |
| Carbuncle | 1 | 0 |

"The great percentage of deaths arising from diarrhœa is the most remarkable feature of the foregoing table; but on this subject I have not now time to enlarge, though I hope to investigate it closely at same future period. It is to be regretted that a more exact system of registration is not generally adopted, especially with regard to this disease; there is no doubt that, at present, many cases of muco-enteritis, tabes mesenterica, and other disorders, are indifferently classed together under the generic name of diarrhœa, and thus any deductions as to its causes, progress, etc., are, to a great extent, rendered impossible. For the accompanying meteorological report I am indebted to the courtesy of Mr. White.

| | *Sept.* | *Oct.* | *Nov.* |
|---|---|---|---|
| | Deg. | Deg. | Deg. |
| Barometer, maximum | 30·190 | 30·250 | 30·290 |
| " minimum | 29·410 | 29·205 | 29·150 |
| " mean | 29·740 | 29·760 | 29·820 |
| Temperature, maximum | 78·5 | 62·5 | 55·1 |
| " minimum | 39·5 | 37·2 | 22·1 |
| " mean | 60·1 | 50·7 | 42·5 |
| Total amount of rain | 1·9 | 3·05 | 1·02 |
| Prevailing direction of wind | S. & SE. | S. & NW. | NW. |
| Mean amount of ozone (Schonbein, &c.) | 8 | 7·5 | 6 |
| Number of deaths | 139 | 112 | 101 |
| " in 1855 | 132 | 117 | 101 |

"Amongst the deaths there were two from aneurism, five from cancer, and one from natural decay, at the great age of 101."

*Burton-on-Trent.*—Dr. Thomson writes: "The past quarter has, generally speaking, been healthly; the most prevalent disease being diarrhœa, but seldom in a severe form. In country places there is one cause of diarrhœa about this time of the year, and, indeed, during the winter, which is not generally noticed. It is the continued use of fresh pork in a family, subsequently to the killing of a pig. After a pig has been killed, there is an unusually abundant supply of animal food, and that not in its most digestible form; consequently diarrhœa, to a greater or less extent, is so frequently consecutive, that I almost invariably put the question as to the fact, when seeing cases of the disease during the winter."

*Oswestry.*—Mr. Cartwright says: "The cases of remittent fever approached much to an 'intermittent,' being decidedly aguish in character. Fever of a continued kind, classed under 'typhus,' commenced, for the most part, with ulcerative stomatitis, bleeding from gums and nose, and requiring support early; *large* purpurous spots appeared in two cases. The mortality was about 1 in 12."

*Alford.*—Dr. West says: "I think rheumatic complaints are, and have been, unusually prevalent for several weeks. I have had several cases of apparent acute rheumatism, which have proved to be periostitis; ending in suppuration and exfoliation of bone. In one case, the knee and ankle joints were affected together, and one wrist; suppuration took place near the knee and near the ankle—the wrist escaping. I think I was enabled to trace the affection to the direct application of wet and cold. In another case in which suppuration took place on the upper part of the tibia, the patient (a little boy) died suddenly, after suffering great pain in the abdomen."

*Staveley.*—Mr. Thorpe says: "There have been a good many cases of fever, but of a mild character, with the exception of a few in a dirty, ill ventilated situation. I have noticed that patients have recovered but indifferently after childbirth; and two cases of puerperal fever have occurred; one a very bad case. Carbuncle has been very general for a long time. With regard to cattle, there have been many cases of milk fever in the neighbourhood. Potatoes have been very much diseased."

*Gainsborough.*—Dr. Mackinder writes: "The prevailing disease this season has been typhoid fever, which almost deserves the epithet epidemic; and, though generally of a mild character, it has been fatal in a few cases. In my last report I referred to a few cases of severe typhoid which had occurred in a very circumscribed but elevated district—the southern extremity of a village four miles hence. The poor woman last spoken of died, and just before her death the mother of a large family, living in an ill ventilated cottage, a couple of hundred yards distant, was seized with the fever, and must have sunk under it had it not been for the kind and unremitting attention of a Crimean nurse, the sister of a local baronet. During the illness of this patient it was discovered that a pestilential effluvia arose from an adjoining well, which had long been the receptacle of dead dogs, cats, etc. This being closed, the fever disappeared. About a mile from the town I have had a severe case of English cholera, the secretion of urine being arrested for thirty-six hours."

The following Meteorological Report is furnished by Mr. Dyson:—"The barometrical pressure ranged very high in October, the maximum being upwards of 30.5 inches on the 25th. The lowest was 29 inches on the 23rd September. The month of October was wet, especially in the first fortnight; November generally clear and fine. The amount of rain which fell in Sept. was 2.84 inches; in Oct., 2.53 inches; and in Nov., 1.32 inches. November was remarkable for the absence, and the two preceding months for a free development, of ozone. On the night of the 23rd November, the temperature fell 20°—from 52° to 32°—and the winter set in with unusual severity at that early period, and continues up to this date (Dec. 3rd). The coldest night was the 2nd in Dec., when the *minimum* thermometer indicated 20°, and 21° the following morning at nine o'clock. Fully 6 inches of snow fell on the night of the 26th Nov.; and the greater portion remains on the ground. The highest temperature was 74° on the 1st Sept., and the lowest 20° on the 29th Nov., so that the range of temperature in the quarter was 54°."

*Liverpool.*—Mr. Bickerton furnishes the following statements: "Sept. 13. There has been little change in the weather, but the health of the town continues in a very satisfactory state; the deaths averaging seventy-two less than in the corresponding week of last year. There have been registered during the past seven days from scarlet fever 6 deaths; measles, 4; small-pox, 3; diarrhœa, 64; fever, 4; disease of lungs, 48; disease of brain, 9; convulsions, 1; diseases of the stomach, 3; dentition, 4; old age and natural decay, 30.

"Sept. 27. Two hundred and forty deaths were registered during this week, being 21 more than last week. Measles caused 2; small-pox (not vaccinated), 2; typhus, 16; diarrhœa, 41; scarlet fever, 14; hooping-cough, 4; syphilis, 3; disease of lungs, 56.

"During the quarter ending September 27, 1856, 3,018 deaths have been registered in the borough, including 152 on which inquests have been held. The number is 321 less than the average mortality of the same quarter of former years, excluding epidemic cholera. Zymotic diseases caused 1,024 deaths, the average being for the same quarter of former years, without cholera, 1,080. Of this number 537 were from diarrhœa (the average being about 450); scarlet fever, 124; typhus, 90; measles, 75; hooping-cough, 55; small-pox (of which two only had been previously vaccinated), 28; dysentery, 25; tracheitis, 22; cholera, 21; syphilis, 16; and mumps, 2.

"Oct. 4. The health of the town continues satisfactory. The mortality has been 20 per cent below the average; the number of deaths registered, 192, being a smaller number than in any of the previous nine weeks. Scarlet fever caused 13 deaths; measles, 4; small-pox, 1 (not vaccinated); typhus, 11; diarrhœa, 24; cholera, 1; syphilis, 1; mumps, 1; disease of lungs, 50.

"Oct. 11. The mean temperature was 63½°. One hundred and eighty-five deaths were registered; or 37 less than the corrected average for previous eight years. Scarlet fever caused 22 (highest for the last seven months); measles, 1; small-pox, 3 (unvaccinated); diarrhœa, 14; English cholera, 1; typhus, 7; syphilis, 2; disease of lungs, 44, including 18 from phthisis. One hundred and eleven were under the age of 5 years; eleven between 5 and 15; forty-nine between 15 and 60; fourteen above 60.

"Oct. 18. The health of the town has been favourable; 203 deaths have occurred, against the average of 221 for previous nine years. The temperature has been high during the week.

"Nov. 22. A considerable decrease has taken place in the mortality; only 192 deaths registered. Scarlet fever caused 17 deaths; measles, 5; small-pox, 3 (one only vaccinated); hooping-cough, 7; typhus, 3. Disease of lungs caused 70 deaths; 22 from phthisis pulmonalis; one-half the deaths occurred below 5 years of age, thirty-five were above 60 years old.

"Nov. 29. Two hundred and nine deaths were registered, or about the average of previous years. From scarlet fever there were 13 deaths; measles, 10; small-pox, 3 (not vaccinated); hooping-cough, 3; typhus, 6; tracheitis, 6. Diseases of lungs caused 80 deaths; 55 from bronchitis and inflammation; 20 from phthisis; 119 were males; 90 females: 111 were below 5 years old; 13 between 5 and 15; 72 between 15 and 60, and 13 above 60. Temperature very variable; highest was 53° 2′ 10″, Monday: the lowest, 31° 6′ 10″ on Saturday.

*Wigan.*—Mr. Cox says: "The only important cases this quarter have been those of diarrhœa. Those occurring amongst adults mostly yielded to ordinary treatment; but children (especially infants) have suffered more severely, and several have been carried off. The symptoms in many cases were those described by Rokitansky as characteristic of 'softening of the stomach.'"

*Bolton-le-Moors.*—Mr. Pendlebury writes: "From several sources I have ascertained that the past quarter has not been an over-busy time for the 'doctors.' Cases of typhus fever have been perceptibly fewer and milder than at this season generally. An ephemeral type of fever has been prevailing of late, and has required early tonics. The mortality amongst children suffering from pneumonia and bronchitis (which have been particularly rife) has been rather high. Hooping cough is rather on the increase. Two or three cases of small-pox have occurred in unvaccinated children, one of which was fatal. The prevailing winds have been N. and N.W., and latterly attended with a clear and frosty air in lieu of 'chill November's surly blast'; and which may account for the comparatively few cases of articular rheumatism at this time."

*West Auckland.*—Mr. Todd says: "During the present quarter the weather has been remarkably wet,—hardly one dry day during the whole of September and October. All the corn in this neighbourhood is unsound, and the potato disease is general and worse than in any former year. Some cases of mild remittent fever were treated during the month of September. One case of confluent small-pox occurred after vaccination. The patient, a female, aged 40, in the seventh month of pregnancy, visited a friend in Darlington, whose daughter was recovering from small-pox. Ten days after this exposure she suffered from rigors, succeeded by fever, and the eruption appeared on the twelfth day from exposure to the contagion. The patient was convalescent in a month from the attack, and was delivered of a very healthy female child six weeks after her recovery from small-pox. Hooping cough has been very prevalent; in many cases complicated with bronchitis and pneumonia. Continued fever of a mild form—without complications, save in some instances slight gastro-enteric affection—has prevailed during the quarter. There have been no deaths—indeed the mortality from epidemic diseases has been very slight. The weather during the last month has been dry, cold, and frosty, and we have now a good covering of snow."

*Lerwick.*—Dr. Spence says: "The weather during the first two months of the quarter was unusually mild: the winter commenced with some severity in the beginning of November, the first snow fell on the 9th. The most important disease has been small-pox, of which there have been thirty-two cases and one death. During September it seemed to be decreasing; but in the beginning of October a person coming from Liverpool was attacked with the disease in the steerage of a coasting vessel, carrying a number of passengers, and this contributed much to the spread of the disease. In an adjoining parish there was one case of small-pox which proved fatal. There was one case of typhoid fever in October, which terminated fatally. The average age of the persons who died under medical treatment was forty-five, only one being under five. The causes of death were as follows: typhoid fever, 1; small-pox, 1; bronchitis, 1; disease of the heart, 1; chronic disease of liver, 1; debility from old age, 1."

---

### STRAY NOTES.

SANITARY REFORM. "What a yet unspoken poetry there is in that very Sanitary Reform! It is the great fact of the age. We shall have men arise and write epics on it, when they have learnt that 'to the pure all things are pure,' and that science and usefulness contain a divine element, even in their lowest appliances." (Kingsley.)

THE INDELICACY OF IMBIBING IMPURE AIR. Persons fond of frequenting unwholesome crowds, ought, says Trotter, to be informed, that nothing is so indelicate as to breathe *respired air*, or that exhaled from the lungs of other people: to drink of the same cup is the height of politeness compared with this custom.

